# Thromboinflammation in Myeloproliferative Neoplasms (MPN)—A Puzzle Still to Be Solved

**DOI:** 10.3390/ijms23063206

**Published:** 2022-03-16

**Authors:** Vikas Bhuria, Conny K. Baldauf, Burkhart Schraven, Thomas Fischer

**Affiliations:** 1 Institute of Molecular and Clinical Immunology, Otto-von-Guericke University Magdeburg, 39120 Magdeburg, Germany; vikas.bhuria@med.ovgu.de (V.B.); conny.baldauf@med.ovgu.de (C.K.B.); thomas.fischer@med.ovgu.de (T.F.); 2 Health-Campus Immunology, Infectiology, and Inflammation, Medical Center, Otto-von-Guericke University Magdeburg, 39120 Magdeburg, Germany; 3 Center for Health and Medical Prevention—ChaMP, Otto-von-Guericke University Magdeburg, 39120 Magdeburg, Germany

**Keywords:** neutrophils, integrins, MPN, inflammatory cytokines, thromboinflammation

## Abstract

Myeloproliferative neoplasms (MPNs), a group of malignant hematological disorders, occur as a consequence of somatic mutations in the hematopoietic stem cell compartment and show excessive accumulation of mature myeloid cells in the blood. A major cause of morbidity and mortality in these patients is the marked prothrombotic state leading to venous and arterial thrombosis, including myocardial infarction (MI), deep vein thrombosis (DVT), and strokes. Additionally, many MPN patients suffer from inflammation-mediated constitutional symptoms, such as fever, night sweats, fatigue, and cachexia. The chronic inflammatory syndrome in MPNs is associated with the up-regulation of various inflammatory cytokines in patients and is involved in the formation of the so-called MPN thromboinflammation. JAK2-V617F, the most prevalent mutation in MPNs, has been shown to activate a number of integrins on mature myeloid cells, including granulocytes and erythrocytes, which increase adhesion and drive venous thrombosis in murine knock-in/out models. This review aims to shed light on the current understanding of thromboinflammation, involvement of neutrophils in the prothrombotic state, plausible molecular mechanisms triggering the process of thrombosis, and potential novel therapeutic targets for developing effective strategies to reduce the MPN disease burden.

## 1. Introduction

The classic Philadelphia chromosome-negative myeloproliferative neoplasms (MPN) are clonal hematopoietic stem cell disorders characterized by the excessive production of myeloid progenitor cells, resulting in the accumulation of high numbers of mature blood cells. The World Health Organization has classified several diseases under the term MPN; however, polycythemia vera (PV), essential thrombocythemia (ET), and primary myelofibrosis (PMF) are considered the main set of Philadelphia chromosome-negative MPNs [1,2,3]. PV patients primarily show increased formation of red blood cells, whereas ET patients show strongly elevated numbers of thrombocytes, while PMF, among other features, is characterized by fibrosis of the bone marrow and splenomegaly. Genomic analysis revealed the occurrence of a somatic activating point mutation in the JAK2-kinase at amino acid position 617 (JAK2-V617F) in the majority (95%) of PV patients and in approximately 50% of ET and PMF patients [4,5]. In addition to the JAK2-V617F mutation, activating point mutations in the thrombopoietin receptor MPL (MPL-W515L/K) and a few other MPL exon 10 mutations have been discovered in 4% to 6% of ET and PMF, but not in PV patients [6,7]. In most of the remaining ET and PMF patients without mutated JAK2 or MPL, a spectrum of mutations within the Calreticulin (CALR) gene has been identified and is found in 25% of ET and PMF patients [8,9]. Nevertheless, there are also so-called triple-negative MPN, characterized by a lack of mutations in JAK2, MPL, and CALR. In these patients, most of the mutations that have been described occur in epigenetic regulator genes (TET2, ASXL1, DNMT3A, EZH2, and IDH1/2), genes of messenger RNA splicing (U2AF1, SF3B1, SRSF2, and ZRSR2), and DNA repair (TP53). Of note, these mutations are known to occur in other myeloid malignancies as well [9].

The expected annual incidence rate of MPN is approximately 2 cases per 100,000 of the population [10]. Interestingly, newer studies report incidence rates of up to 5 cases per 100,000 [11]. Due to its chronic course, the prevalence rates of MPN are considerably higher. In PV and ET, the prevalence was estimated to be 30 cases and 40 cases per 100,000, respectively [12]. Thus, PV and ET may not be considered as rare diseases, but rather as relatively common hematologic malignancies. However, there are little data on the prevalence of PV and ET and of other MPNs in the current literature. The clinical hallmark of MPN is abnormal blood counts; however, many patients experience an inflammatory syndrome presenting as fever, night sweats, cachexia, itching, and elevated levels of C-reactive protein (CRP) [13]. Splenomegaly, which is most pronounced in the late stages of the disease, and leukocytosis account for some of the most serious signs of myelofibrosis (MF). In addition, the major causes of morbidity and mortality in MPNs are venous and arterial thrombosis and bleeding, including deep vein thrombosis (DVT), myocardial infarction (MI), and strokes [14,15,16,17].

The clinical picture and the prognosis of patients are strongly influenced by the genetic signature. In PV, an incidence of up to 23.4% thrombotic events (arterial (16%) and venous (7.4%)) at diagnosis has been reported [18]. During the follow-up after diagnosis, the incidence of non-fatal and fatal thrombotic events was 10.3% and 4.5%, respectively [18]. Interestingly, in PMF, a recent meta-analysis found that CALR-mutated patients displayed a lower risk of splenomegaly (OR 0.47, 95% CI 0.29–0.78) and thrombosis (OR 0.52, 95% CI 0.29–0.92) when compared with JAK2-mutated patients [19]. Nevertheless, the risk of thrombosis in CALR-mutated patients is considerably higher than that in the non-MPN-patient population. Even though disturbances in the blood flow and increased viscosity are among the triggering factors for the development of arterial and venous thrombosis, the underlying pathophysiology and chronology of the increased thrombotic risk in MPN have remained an enigma until now. Various studies have suggested that high levels of cytokines/chemokines secreted by clonal and non-clonal cells create a chronic proinflammatory state that contributes to MPN disease pathogenesis and initiates or perpetuates thrombosis [20,21]. Moreover, our previously published data suggest that the aberrant activation of leukocyte immune functions, e.g., integrin activation, promotes pathological thrombus formation in the vasculature [22]. The observed upregulation of inflammatory cytokines has also been implicated in the formation of the MPN thrombo-inflammatory state [13].

The term “*thrombo-inflammation*” has been described as the pathological responses within the vasculature following blood vessel injury, invasion by pathogens, or non-infectious (sterile inflammation) inflammatory triggers [23] leading to acute organ damage. MPN clones stimulate systemic inflammation by elevating various proinflammatory cytokines (for an overview, please see Table 1) through a complex process, which is not yet understood completely. Given the high incidence and mortality rate of thrombotic events in MPN, there is a need to comprehensively characterize the molecular events underlying inflammation and hemostasis in order to develop new therapies and effective strategies to prevent and treat thrombosis. In this review, we will provide an overview of the inflammation-induced usual suspects, novel candidates, and essential molecular mechanisms leading to the pro-thrombotic state in MPNs.

## 2. Methodology of Literature Search

The preparation of the current narrative review was based on a literature search using suitable keywords related to the topic of interest on several databases to make sure that most of the relevant studies were identified. The databases that were used to search for the studies to write this review were mainly PubMed, Google Scholar, and MEDLINE. For each published article, we explored the full texts and, in addition, we preferred to manually select the other cited publications. All types of published articles, which were written in English, were included and gray literature was excluded. During the crafting of the text, the introduction and conclusion were drafted after the finalization of the other sections. Additionally, to eliminate irrelevant text to the main discourse, the text was reassessed. Likewise, the drafting of the conclusion followed the critical assessment process.

## 3. Inflammatory Cytokines

Inflammation refers to a critical physiologic process of fighting against invading pathogens, injuries, and toxins in an attempt to heal itself through the activation of the host defense system. However, the lingering of inflammation develops into chronic inflammation, keeping the organism in a persistent state of alert. Chronic inflammation may also result in genomic instability, supporting the initiation of neoplasms. There is abundant evidence available that points out the link between chronic inflammation and the progression of MPNs into secondary myelofibrosis or leukemia, both of which carry a worse prognosis [44,45,46,47,48,49].

MPN-associated neoplastic and normal cells are known to secrete numerous proinflammatory cytokines that play an important role in the development of constitutional symptoms, and have been discussed as drivers of disease progression and are involved in the prothrombotic state [50]. Various studies have found widespread elevation in cytokines such as IL-2, s-IL-2R, IL-6, IL-1β, IL-8, and TNF-α, which are associated with MF transformation from PV and ET patients [21,39,51]. Interleukin-1β (IL-1β) is a key regulator of the inflammatory state [52] that, upon deregulation, appears to be directly associated with MPN progression [53]. IL-1 β is secreted by a limited number of cells, such as monocytes, macrophages, and dendritic cells. It mediates systemic inflammation and is involved in stimulating several other proinflammatory cytokines, such as IL-6, TNF-α, and granulocyte colony-stimulating factor (G-CSF) [54,55,56]. IL-1β is also known to be important for homeostasis maintenance, as well as emergency hematopoiesis. Studies by Tefferi and colleagues revealed elevated serum levels of IL-1β in PV (n = 65) and PMF (n = 127) patient cohorts [21,28]. Furthermore, elevated levels of IL-12, IL-15, IP-10, IL-8, IL-2, and s-IL-2R in PMF patients have been correlated with decreased/poor overall survival [21,28,57]. Other very important participating factors, the NLRP3 and AIM2 inflammasomes, have appeared as crucial players in regulating certain hematological pathologies, e.g., myelodysplastic syndrome (MDS), MPNs, cardiovascular risk, and leukemia [58,59]. The inflammasome plays an important role in the development and expansion of HSPCs, as well as their release from bone marrow (BM) into peripheral blood under stress conditions [58,60]. A recent study by Ying Zhou et al. determined that inflammasome-related genes, including NLRP3, were expressed at high levels in the BM cells of MPN patients [61]. Although the role of NLRP3 has been studied comprehensively in the case of MDS [62,63], there is a need for more detailed studies to explore its role in MPN pathophysiology. Moreover, the myeloid cell-secreted cytokine lipocalin-2 (LCN2) or neutrophil granulocyte-associated lipocalin (NGAL) have often been found to be elevated in PV, ET, and PMF patients as compared to healthy controls, suggesting roles in the fibrotic transformation of the bone marrow microenvironment [64,65,66]. This cytokine stimulates the growth of splenic endothelial cells, followed by increased IL8 production by splenic stromal cells [67]. In a murine model study, Kagoya and colleagues determined that JAK2-V617F-positive cells induce LCN2, resulting in DNA strand breaks and apoptosis in neighboring healthy cells through the generation of reactive oxygen species (ROS) [68]. Apart from its role in bone marrow, high LCN2 expression has also been found in response to stress-related signals. In a study by Tefferi et al., elevated plasma levels of IL6 and IL-8 were correlated with the severity of constitutional symptoms in PMF patients [21]. Aside from elevated levels of pro-inflammatory cytokines, anti-inflammatory cytokines, such as IL-4 and IL10, were also found to be increased in MPN cohorts and probably play a role in counteracting the deregulated pro-inflammatory response [33].

Another important cytokine, tumor necrosis factor α (TNF-α), has been seen to be strongly elevated in different MPN subtypes (PV, ET, and PMF) [36,69,70]. Interestingly, increased plasma TNF-α levels were significantly correlated with the JAK2-V617F allelic burden [37]. In many MPN studies, TNF-α has been shown to promote the growth of the malignant clone, whereas the proliferation of the healthy clone was inhibited. However, the differences in the underlying signaling events between the malignant clone and normal cells are not entirely clear [37]. Defective negative regulation of Toll-like receptor signaling has been reported to be involved [71]. In another study, the inhibition of TNF-α receptor 2 (TNFR2) attenuated the clonogenicity of PMF patient-derived CD34+ cells. Conversely, in a retrovirally induced JAK2-V617F-positive BALB/C BM transplantation model, the in vivo blockage of pan–TNF-α did not reduce the disease burden [72]. Together, these results suggest that the involvement of the two distinct TNF receptors, TNFR1 and TNFR1, may exert dichotomic functions in MPN. In our recently published study, we examined the therapeutic efficacy of anti-αTNFR1 and αTNFR2 antibody treatment using the established JAK2-V617F knock-in mouse model. Although αTNFR2 antibody treatment strongly diminished the number of white blood cells, no effect was seen on the hematocrit or on splenomegaly. Interestingly, treatment with αTNFR1 antibody led to an increase in the WBC, mild suppression of the elevated hematocrit, and even reduced splenomegaly [73]. This shows the dichotomic functional aspects of TNFR1 and TNFR2 signaling in MPN, where the inhibition of a single TNF-α receptor exhibits differential biological effects. Although substantial progress has been made in understanding the role of TNF-α-induced inflammation in MPN pathophysiology, various questions remain unanswered.

Furthermore, the excessive secretion of these cytokines fosters the activation of platelets and leukocytes, as well as endothelial cells. This contributes to the formation of mixed leukocyte–platelet aggregates, which release the brakes of coagulation and lead to thrombosis and tissue ischemia [74]. The degree of inflammation is further driven by the cellular interaction between leukocytes, platelets, and endothelial cells. Conventionally, the endothelium is known to regulate vascular permeability and to preclude thrombus formation. However, the discovery of the JAK2-V617F mutation in endothelial cells (EC) showed that leukocytes adhere more tightly to JAK2-V617F-mutated EC compared to normal EC [75,76]. In one of the studies, it was shown that a large number of genes related to inflammation, adhesion, and associated prothrombotic pathways are over-expressed in JAK2-V617F ECs [76]. Additionally, TNF-α, as a secreted proinflammatory cytokine early in the immune response, is considered as a compelling inducer of the immune defense mechanism. It mediates the recruitment of leukocytes and is involved in promoting the procoagulant state via the decreased production of anticoagulant protein C and evoking tissue factor (TF) synthesis [77]. Correspondingly, a study on cecal ligation and puncture (CLP) murine models indicates that activated platelets also adhered aggressively to inflamed endothelial cells and leukocytes, resulting in ‘heterotypic aggregation’ in circulation [78].

Aberrant increases in inflammatory cytokines foster the formation of thrombosis by a multitude of cellular and molecular mechanisms. These include the up-regulation of endothelially expressed VCAM-1 and ICAM-1, integrin activation on leukocytes, recruitment of granulocytes and monocytes, formation of NETosis, platelet activation and aggregation, induction of TF, thrombin expression, and the activation of plasmatic coagulation. The role of particular cytokines in driving these events was recently thoroughly discussed and reviewed by Najem-M and colleagues [41]. Another important candidate, reactive oxygen species (ROS), has been postulated to be engaged as an endogenous ‘inflammatory carcinogen’ in MPNs. The augmentation of the ROS levels, generally referred to as oxidative stress, has been seen as a major concern with regard to the development of cancer, both in solid tumors and in hematological malignancies [79]. Oxidative stress is known to stimulate the redox-sensitive NF-κB pathway and thereby increase inflammation by initiating proinflammatory cytokine and chemokine production [79]. Interestingly, in a study by Moisa et al., it was observed that JAK2-V617F-positive ET patients with previous thrombosis had higher oxidative stress levels than patients without any previous occurrence of thrombotic events [80]. Moreover, in another study of ET patients, the authors investigated the relationship between ROS, chronic inflammation, total antioxidant capacity, leukocytosis, JAK2-V617F mutation, and disease progression to myelofibrosis, and concluded that chronic inflammation and oxidative stress are involved in the induction of ET disease and also in the transformation into secondary myelofibrosis or leukemia [81].

Currently, there is strong interest in the thesis that inflammation is a driving force behind many solid tumors and hematologic malignancies, including MPNs. Moreover, inflammation is also associated with poorer overall prognosis [82,83,84]. Certainly, inflammation has an incredible feature to enable most, if not all, of the essential molecular features of cellular reprogramming and the suppression of immunosurveillance, which are necessary for carcinogenesis. In a large Swedish epidemiologic study, it was demonstrated that chronic inflammation might play a triggering role in the development of acute myelogenous leukemia (AML) and myelodysplastic syndrome (MDS) [85,86]. In another study from Sweden, it was shown that patients with a prior history of autoimmune disease were more susceptible to developing MPN disease [87]. Along this line, it is interesting to note that inflammation-related diseases, such as Crohn’s disease, tend to increase the frequency of the JAK2 46/1 haplotype [88,89]. Moreover, in some of the recent next-generation sequencing studies, it was demonstrated that asymptomatic clonal hematopoiesis is common among adults and is frequently associated with JAK2-V617F mutation [90]. Besides, in an elegant study investigating the life histories of MPN inferred from phylogenies, it was shown that the JAK2-V617F mutation is commonly associated with clonal hematopoiesis, which occurs during childhood, including in utero. Interestingly, the mean latency between JAK2-V617F acquisition and diagnosis was described to be 30 years (range 11–54 years) [91]. Further, it appears that chronic inflammation is indeed indispensable in the transformation of asymptomatic JAK2-V617F mutant clones into apparent MPNs [92,93,94]. However, additional studies are required to precisely characterize the molecular mechanisms involved and to provide unambiguous evidence on the role of inflammation in the induction and progression of MPNs [91].

## 4. Thrombosis in MPN: The Roles of Integrin Activation and of NETosis

As mentioned above, the innate immune system functions as a purging system, where it counteracts invading foreign bodies and orchestrates thrombo-inflammatory responses by employing the blood coagulation cascade, granulocytes, platelets, and endothelial cells. The deregulation of the innate immune response and the resulting hyperactivity of inflammatory cytokines lead to excessive inflammation and injury. In MPN patients, arterial and venous thrombosis is one of the major causes of mortality and morbidity, strengthening the fact that chronic inflammation plays a pivotal role in thrombosis. The cumulative risk of thrombosis may be as high as 35% upon 20 years of observation, which increases sharply after the age of 45–50 years, and is the most frequent cause of death in PV and ET patients. In a prospective study of 1638 patients with PV, Landolfi and colleagues observed that 38% of patients had already experienced a previous thrombosis at the time of diagnosis, and two-thirds of those were arterial events [95]. In ET, thrombosis is also a common event and is particularly frequent in JAK2-V617F patients [96,97]. A study by the German SAL-MPN registry showed that, out of all 455 patients included, 33.6% had suffered from a vascular event. The most frequent events were deep vein thrombosis (31.5%), acute coronary syndrome (27.7%), strokes (19.3%), and splanchnic vein thrombosis (15.2%). Major bleeding events were reported in 8.2% of all patients [98]. Interestingly, in a recent analysis of 1420 patients from the German Study Group for MPN, there was a strong association of impaired kidney function with the incidence of thrombosis. A total of 29% of the patients had a history of thrombosis [99].

**Figure 1:**
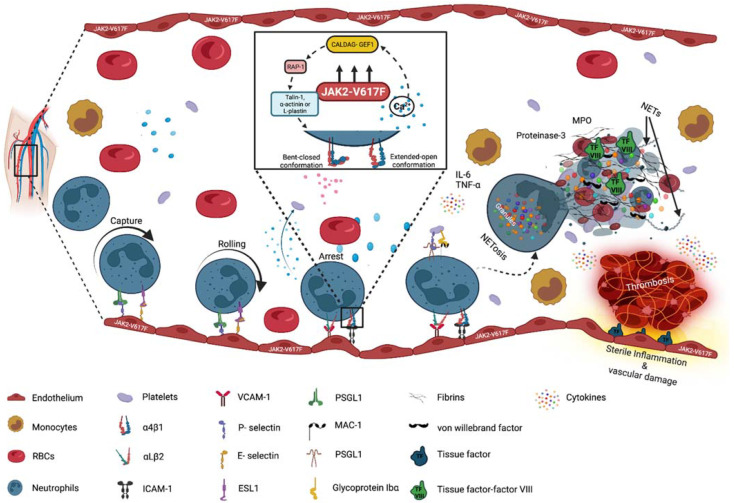
Neutrophil-associated thromboinflammation in MPNs. The constitutive activity of mutated JAK2-V617F kinase increases intracellular Ca^2+^ and supports Ca^2+^ influx followed by CalDAG-GEF1 (calcium- and diacylglycerol-regulated GEFI) activation. This results in the activation of small guanosine triphosphate hydrolase enzymes (GTPases), such as RAS-related protein 1 (RAP1), which further stimulate integrin-binding proteins to facilitate integrin conformational changes in neutrophils. The activation of integrins assists neutrophils in inducing thromboinflammation, which is a multistep process where activated neutrophils, inflammatory cytokines, the aggregation of platelets, and induction of plasmatic coagulation synergize. Initially, under flow conditions, neutrophils interact with endothelium-expressed P- and E-selectin with their respective PSGL-1 and ESL1-ligands, which allow neutrophils to slowly roll along the blood vessel. While rolling, neutrophils are arrested upon the binding of β1 and β2 integrins (VLA-4, LFA-1) to the endothelium-expressed VCAM-1 and ICAM-1. Inflammatory cytokines, such as IL-6 and IL-17, foster this process by up-regulating VCAM-1 and ICAM-1 expression. The release of chemokines leads to chromatin decondensation in neutrophils, which then expel granular proteins, including neutrophil elastase (NE), myeloperoxidase (MPO), and DNA material into the extracellular space to form NETs. The formation of NETs further induces thrombosis by activating plasmatic coagulation and by inducing the aggregation of platelets and erythrocytes. (This figure was created with BioRender.com, assessed on 15 February 2022).

Crucially, the thrombotic risk in PV and ET is far beyond that observed in secondary polycythemia or thrombocythemia, indicating that intrinsic factors over and above the increase in erythrocytes and platelets and in blood viscosity drive the thrombotic risk in JAK2-V617F MPN. Our previously published data suggest that JAK2-V617F-induced aberrant activation of leukocyte β1 and β2 integrins promotes pathological thrombus formation [22,100]. Further, using intra-vital 2P microscopy, we found that JAK2-V617F-induced activation of granulocytes leads to abnormal interaction with the endothelium in a saphenous vein stenosis model (Haage-R and Fischer-T, unpublished data). JAK2-V617F kinase activity induces a change from the inactive bent to the active open conformation of Very Late Antigen-4 (VLA-4, CD49d/CD29, and alpha4 beta1 integrin) and over-activates VLA4 and Lymphocyte Function-associated Antigen-1 (LFA-1, CD11a/CD18, and αLβ2 integrin) in myeloid cell lines and in granulocytes from MPN patients [22]. This results in the up-regulation of β1/β2 integrin-mediated granulocyte adhesion and increased thrombus formation in a deep vein stenosis model. Interestingly, neutralizing antibodies against β1/β2 integrins abrogated the prothrombotic phenotype in JAK2-V617F knock-in mice [22]. This indicates an important role of granulocytes in JAK2-V617F-induced pathological deep vein thrombosis (Figure 1). Similarly, in a recent report by Yago-T et al., anti-β2 integrin antibody treatment suppressed thrombus formation in an IVC model in mice [101].

Correspondingly, the overexpression of the heterodimeric integrin alpha-M beta-2 (αMβ2) molecule CD11b/CD18 has been seen on the neutrophils and monocytes of patients suffering from PMF, particularly in JAK2-V617F-positive patients [102]. The observed upregulation of these factors has been implicated in the formation of increased numbers of circulating platelet–neutrophil (PNC) and platelet–monocyte (PMC) complexes in JAK2-V617F-positive disease. In an arterial thrombosis study by Dhanesha and colleagues, it was observed that myeloid cell-specific integrin α9^−/−^ knock-out mice (α9^fl/fl^LysMCre^+^) were less vulnerable to thrombosis in ferric chloride and laser injury-induced thrombosis mouse models [103].

Another element called neutrophil extracellular traps (NETs), comprising a network of extracellular fibers and DNA-encompassing assemblies from neutrophils, has been constantly reported in MPN patients (Figure 1). Generally, NETs play a crucial role in encountering infection and in innate host defense mechanisms; however, NETs also mediate sterile inflammation [104,105]. During NETosis, neutrophils decondense their DNA and, together with nuclear contents, such as histones, myeloperoxidase (MPO), and elastase DNA, are released to the extracellular space. NETs also have a direct effect on platelet functionality.

In one of the studies by Elaskalani et al., it was determined that cell-free NETs, obtained from LPS-stimulated neutrophils could promote platelet aggregation when incubated together with human platelets [106]. The platelets’ surface expression of CD62 and phosphatidylserine (PS) was also increased, indicating NET-mediated intracellular signaling activation in platelets [106]. These results strongly suggest that cell-intrinsic effects of neutrophil function play a crucial role in thrombosis. As mentioned above, thrombosis is one of the major complications in MPN patients, which could be associated with increased NETosis, but the underlying mechanisms are still not clear. Until now, only a limited number of studies have been performed to understand the contribution of NETs to the procoagulant activity observed in MPNs. An in vivo mouse model study by Wolach et al. demonstrated that increased JAK2 kinase activity is concomitant with NET formation in MPNs, and the use of the JAK inhibitor, ruxolitinib, abrogated thrombus formation [107].

Little is known about the contribution of platelets to the prothrombotic state associated with JAK2-V617F-induced MPN. Experimentation using JAK2-V617F-positive mice to investigate the role of platelets in various thrombosis models has provided heterogeneous results: In ET, the aberrant activation of the PI3 kinase/Rap1/integrin αIIbβ3 pathway leading to ex vivo platelet hypo-reactivity has been described [108]. A study by Hobbs and colleagues in a knock-in ET mouse model found that JAK2-V617F mutation initiates inherent changes in the formation of platelets from megakaryocytes and also alters their reactivity [109]. Furthermore, in a FeCl_3_-induced thrombosis model, instability was observed in swiftly aggregated platelets [110]. Additionally, Hauschner-H et al. observed that platelets from Calreticulin-mutated ET patients were less reactive to ADP (adenosine diphosphate) stimulation than control or JAK2-mutated platelets [111]. A study by Etheridge et al. revealed that, despite having significantly higher platelet counts than the control group in JAK2-V617F knock-in mice, thrombosis was severely attenuated, and no change was found in platelet activation and aggregation in response to agonists. Intriguingly, JAK2-V617F expression had no major effect on platelet function; however, a series of elegant experiments, including bone marrow transplantation assays, showed that JAK2-V617F-positive endothelial cells (ECs) contribute greatly to dysfunctional hemostasis by inducing an acquired von-Willebrand syndrome (AVWS) phenotype [112].

Red blood cells (RBCs), which normally comprise 40–45% of the volume of blood components (so-called hematocrit), were consistently seen to be increased in PV patients. Many studies have revealed the link between increased hematocrit and associated thrombotic risk. However, the thrombotic risk in MPN is far in excess of that observed in patients with increased hematocrit due to secondary causes, e.g., pulmonary disease. Interestingly, it has been reported that JAK2-V617F activates Lu/BCAM-mediated red cell adhesion in PV through an EpoR-independent Rap1/Akt pathway. This mechanism was suggested to play a critical role in initiating abnormal interaction between circulating and endothelial cells in patients with PV [113]. A clinical study published in 1978 was the first to claim the connection between increased hematocrit and thrombotic risk in PV patients [114], which was subsequently confirmed by several other clinical trials [115]. Moreover, in an in vivo study, it was revealed that elevated RBCs increase the rate of platelet accumulation and thrombus growth at the site of vessel injury [116].

Overall, these observations strengthen the view that, in JAK2-V617F-positive MPN, dysregulated activation of integrins on neutrophils/monocytes, platelets, and erythrocytes leads to abnormal integrin-mediated interaction of circulating blood cells with endothelial cells. However, the precise underlying regulatory and molecular mechanisms involved in the prothrombotic state and in subsequent thrombus formation are still unclear.

## 5. Signaling: JAKs and Leukocyte Integrins

The constitutive activation of the JAK/STAT pathway plays a key role in MPN pathogenesis [117,118]. Conventionally, a family of four cytosolic tyrosine kinase genes, JAK1, JAK2, JAK3, and TYK2, exists in the human genome, wherein the translated JAK proteins further interact with seven different STAT proteins to mediate distinctive responses on transcriptional regulation [119]. The growth factors erythropoietin (EPO), thrombopoietin (TPO), and granulocyte-colony stimulating factor (GCSF), through their corresponding receptors erythropoietin receptor (EPOR), thrombopoietin receptor (MPL), and granulocyte-colony stimulating factor receptor (G-CSFR), drive the production of erythrocytes, platelets, and granulocytes, respectively, via the JAK/STAT signaling pathway. The activated JAK/STAT signaling cascades are also involved in many immunoregulatory processes, cellular metabolic activity, differentiation, apoptosis, DNA damage response (DDR), and in direct or indirect transcriptional activation in the normal cell population [120,121]. There is also a clear understanding that the JAK/STAT pathway plays an essential role in oncogenesis [122,123,124]. In addition, JAKs are also involved in the activation of various other signaling pathways, including the mitogen-activated protein kinase (MAPK), the phosphatidylinositol-3′-kinase (PI3K), and the AKT/mammalian target of rapamycin (mTOR) signaling pathways [125,126,127].

Apparently, mutations in JAK2, CALR, and MPL activate JAK2-STAT and several other interconnected pathways in a cytokine and receptor-independent manner [128]. As discussed above, inflammation is a serious complication in MPN, which elicits the site-specific infiltration of leukocytes, thereby inducing thrombus formation. During the recruitment of leukocytes, integrin-mediated arrest on the endothelium is a central step encompassing a course of adhesive events, including increases in integrin affinity and avidity. A plethora of signaling processes, known as inside–out signaling, are involved in the endogenous regulatory process of integrin activation [129,130,131,132]. These include PI3K, phospholipase C (PLC), calcium diacylglycerol guanine nucleotide-exchange factors (CalDAG-GEFs), small GTPases, adhesion- and degranulation-promoting adaptor protein (ADAP), and others. Amongst these, regulation by small GTPases has been the best studied until today. Small GTPases are molecular on/off switches in cells that regulate the activity of effector proteins by alternating between an inactive GDP-bound off state and an active GTP-bound on state. Ras GTPase is the predominant member of the small GTPase family, and Rap1, a close member of Ras-family GTPases, has recently been in focus for playing an essential role in cellular transformation. There is an extensive body of literature highlighting Rap1 as a central intracellular signaling node controlling the activation of integrins in leukocytes [133,134]. The interaction of Rap1 with Talin1, an adaptor molecule that binds to the intracellular domain of integrins and activates them, has been determined in granulocytes and platelets [135]. A study by Montresor and colleagues showed that JAK protein kinases, along with Rho and Rap small GTPases, mediate LFA-1– and VLA-4–integrin activation and GTPase-dependent lymphocyte trafficking [136]. A similar observation was made in a study by our group, wherein the adhesion activity of β1 and β2 integrins was seen to be upregulated in mice and human JAK2-V617F-mutated granulocytes, as well as in JAK2-V617F-overexpressed 32D murine myeloid cells [22,137]. Additionally, it was observed that PI3K and CalDAG-GEFI regulated the increase in RAP1 activity and increased granulocyte static adhesion on VCAM-1 and ICAM-1 [22], showing the role of Rap1 as an effector, as well as upstream regulator, of Ca^2+^ signaling.

The deficiency of CalDAG-GEFI in neutrophils has been associated with impaired slow rolling, adhesion, and directionality of migration [138,139,140]. Furthermore, despite the regulatory behavior of migration in immune cells, Rap1 signaling is also critical in modulating the Ca^2+^-dependent regulation of Toll-like receptor (TLR) signaling [141,142,143]. In another study, murine neutrophils lacking CalDAG-GEF1/Rap1 were deficient in firmly adhering to the stimulated endothelial venules. Moreover, their migration behavior towards inflammatory sites was severely compromised [139]. On the contrary, increased cell migration has been seen in chronic lymphocytic leukemia (CLL) cells upon the high protein expression of CalDAG-GEFI/Rap1 [144]. These data underline the significance of Rap1 in modulating Ca^2+^-dependent immune responses. Therefore, it is postulated that overactivated CalDAG-GEFI/Rap1 signaling could be an important mechanism underlying the atypical function of integrins in neutrophils. This leads to abnormal leukocyte–endothelium interactions contributing to pathogenic thrombosis in MPNs.

## 6. Extracellular Vesicles (EVs) and Circulating Endothelial Cells (CEC) in Thrombogenesis

Since there is inflammation-associated thrombotic risk in MPN patients, other important factors, such as EVs and CEC, are in the limelight. EVs are known as a heterogeneous population of nano to micro-sized lipid bilayer particles released by most cell types. The EVs derived from blood cells have been validated for their potential role in MPN disease regulation, specifically their orchestration between the MPN clone and the microenvironment [145,146]. In many studies, blood cancer cell-derived EVs have been seen in the functional regulation of important activities, such as chemoresistance, immune regulation, and coagulation cascade. A study by Anna Fel and colleagues analyzed the serum EVs’ proteome in PV patients and observed many deregulated and elevated immune and inflammatory proteins (HPSE, CAMP, LYZ, SELL, and LTF), together with high concentrations of procoagulant and angiogenic agents [147]. In another study on MPN patients by Chaepentier et al., the evaluation of circulating platelets micro particles (PMPs) and their functional relationship with regard to procoagulant activities was analyzed. The study concluded that JAK2-V617F patients had more circulating PMPs and higher procoagulant activities than CALR-mutated or triple-negative ET patients, indicating the key role of circulating particles in higher thrombotic risk induction [148]. This suggests that EVs play an important role in the prothrombotic state.

Since endothelial cells play a vital role in controlling hemostasis and thrombosis, a number of CRCs have been seen in different pathological conditions, which may reflect injury to the endothelium [149]. CECs display a rare peripheral blood cell subpopulation from the mature EC and are found to be correlated with venous thromboembolism in MPN patients. The study by Torres et al. showed elevated levels of CD142+ and CD54+ CECs in the MPN patient group. Additionally, a positive correlation was observed between CD54+ CECs and the antithrombin levels, as well as between the CD142+ CEC counts and the number of thrombotic events [150]. In another study by Treliński and colleagues, the median values of the CECs were found to be markedly higher in PV patients, together with WBC counts of more than 8.7 × 10^9^/L. Besides, the group identified an increase in coagulation activity in both ET and PV patients, but found no difference in the levels of coagulation markers in relation to the JAK2-V617F mutational status [151]. Overall, the above-mentioned studies demonstrated that EVs and CECs play a role in the underlying mechanisms that are involved in the process of thrombosis.

## 7. Current Therapeutic Strategies to Reduce the Prothrombotic Risk

The risk of recurrent arterial and venous thrombosis remains lifelong in all MPN subtypes, but there is an uncertainty in the antithrombotic strategy and treatment. In low-risk PV patients, a low dose of aspirin and phlebotomy to maintain the hematocrit is recommended, while, in high-risk patients, cytoreduction is usually started in addition to aspirin and phlebotomy [152,153]. According to the European Collaboration on Low-Dose Aspirin in Polycythemia Vera (ECLAP) study, aspirin is considered as a successful treatment for thrombosis prevention in PV disease [95]. There are also some reports recommending aspirin twice a day for patients with high risk of arterial thrombosis [154,155,156]. Similarly, in ET patients clinical management to prevent thrombosis takes into account the particular risk profile of the patients: (a) observation only for *low-risk* patients; (b) low-dose aspirin without cytoreduction in *intermediate-risk* patients; and (c) low-dose aspirin together with cytoreduction in *high-risk* patients is considered as the standard of care [157].

Philadelphia-negative MPN have been treated with nonspecific cytoreductive drugs, such as hydroxyurea (HU), pipobroman, or busulfan, to reduce the thrombotic risk [16,158,159,160]. After conducting a multitude of randomized and non-randomized trials, HU, a ribonucleotide reductase inhibitor, has been established as a common first-line chemotherapy agent in MPN [154], but the mechanism by which it decreases the risk of thrombosis remains obscure. In a recent study using a JAK2-V617F mouse model, it was shown that HU treatment reduced thrombosis and abrogated the interaction between leukocytes and JAK2-V617F-expressing endothelial cells by inhibiting endothelial P-selectin expression [75].

A well-known selective JAK inhibitor, Ruxolitinib, has shown significant effectiveness in controlling hematocrit and constitutional symptoms in PV or PMF patients who are intolerant or have developed resistance to hydroxyurea [161,162,163,164]. On the other hand, a recent meta-analysis of clinical trials using Ruxolitinib failed to prove that the use of the JAK inhibitor reduces thrombosis in PV patients in comparison to the previously available therapy [165]. However, a previous meta-analysis of clinical trials using Ruxolitinib as a treatment reported a substantial decrease in thrombosis (risk ratio 0.45, 95% confidence interval (CI) 0.23–0.88) [166]. Ruxolitinib is known to target JAK1 and JAK2 and inhibits serum inflammatory cytokine secretion, thereby reducing symptoms such as fever, night sweats, itching, and fatigue, and splenomegaly [31]. It was shown to improve the quality of life and, to some extent, the overall survival in MPN patients. A study by Deininger et al. determined that long-term treatment of MF patients with Ruxolitinib reduces the JAK2-V617F allele burden and, in some patients, it was possible to achieve complete or partial molecular remission [167].

Interferon-alpha (IFNα), an immune-modulating cytoreductive drug with a specific mode of action, has opened a new perspective for treating MPN patients [168,169]. Interferon α-2 has been evaluated successfully in the therapy of PV and ET patients [170]; however, the exact mechanism of its selective suppression of the MPN clone is still not known. In a preclinical study, it was demonstrated in a JAK2-V617F mouse model that IFNα was able to eliminate PV-like disease in a selective manner by targeting JAK2-VF617F hematopoietic stem cells over JAK2-WT cells [171,172]. However, despite its clinical efficacy, IFNα is correlated with a substantial toxicity profile. In some clinical trials, it was reported that the patients had developed side effects, such as fever, fatigue, nausea, and vomiting [170,173,174]. Considering these limitations, PEGylated interferon alfa-2a and 2b, with improved tolerability, have been synthesized. In a study of PV and ET patients, PEGylated interferon α-2a was investigated as a second-line treatment for patients who were previously refractory and/or intolerant of HU, where the cumulative incidence of major vascular events at one year was 2% (95% CI, 1–8%) and at 2 years was 5% (95% CI, 2–15%) [175]. Similarly, in a study involving CALR-mutated ET patients, pegylated IFNα reduced the CALR variant allele fraction from 41% to 26%, whereas two patients achieved a complete molecular remission response for more than a year after the termination of IFN treatment, suggesting long-standing treatment-free hematological responses in CALR mutated patients [176].

In search of molecularly targeted therapies against thrombosis, anti-integrin monoclonal antibodies could be envisioned to interfere with thrombotic pathophysiology in MPN. This idea is based on a number of reports on the pathologic integrin-mediated interaction of leukocytes, red blood cells, and platelets with the endothelium, as discussed above. In a recent study by our group using the JAK2-V617F knock-in mouse model, it was shown that the neutralization of VLA-4 and β2 integrins completely abrogated thrombosis in a stenosis model of inferior vena cava [22]. These data suggest that leukocyte integrins, potentially VLA-4 and LFA-1, may serve as valuable targets in the therapeutic intervention of thrombosis in MPN in the future. However, in a phase II trial, an anti-CD18 monoclonal antibody developed by Genentech (San Francisco, WA, USA) to treat heart attacks failed to meet its primary objective of improved coronary blood flow [177]. In addition, another anti-CD18 antibody, Leukarrest, developed by ICOS (Bothell, WA, USA) failed to treat ischemic strokes and heart attacks [177]. Despite these apparent negative results, we hypothesize that the outcome in the treatment of thrombotic JAK2-V617F-positive MPN would have been different, because of the mutation-inherent overactivation of LFA-1, (CD11a/CD18) [22]. Thus, in our view, a pilot trial to test this hypothesis is warranted. Along this line, blocking agents for overactivated VLA4 may alternatively be evaluated as therapeutic targets to treat thrombosis in MPN. A proof of concept that targeting integrins is indeed effective and safe in the treatment of thrombosis is provided by αIIbβ3 integrin antagonists. There are three agents, two small molecules and an antigen-binding antibody fragment, that have been approved as integrin-targeting drugs for acute coronary syndrome and thrombotic cardiovascular events [178,179]. Moreover, in an in vivo study, it was shown that anti-M2 antibody targets the interaction between leukocyte Mac-1 and platelet GPIbα, thereby inhibiting thrombosis [180]. Nevertheless, Natalizumab, an anti-α4 monoclonal antibody targeting α4β1 and α4β7 integrins, has been approved for patients with multiple sclerosis (MS) and inflammatory bowel disease [179]. Overall, as also discussed in Chapter 4, in many patients, integrin-based therapeutics have shown clinically significant benefits that further disseminate medical interest in the development of novel integrin inhibitors.

## 8. Conclusions and Future Direction

Thrombo-inflammation has now been acknowledged as an integral component of MPN pathogenesis. The suppression of arterial and venous thrombotic risk is still one of the major goals in managing MPN diseases. Although significant advancements have been made in understanding the inflammatory processes underlying MPN pathophysiology, a multitude of questions remain unanswered. Despite the ground-breaking introduction of targeted therapies in MPNs over the last decades, existing therapies are insufficient to eliminate the malignant clone and fail to durably abrogate concurrent JAK2-V617F or CALRmut-induced inflammation, including the pro-thrombotic risk. Furthermore, during inflammation, certain proinflammatory cytokines are involved in integrin activation, thereby endorsing the abnormal adherence of blood cells to the endothelium. The induction of inflammation is stimulated by both the intrinsic activation of the inside–out signaling of integrins in leukocytes and by high pro-inflammatory cytokine levels in peripheral blood, and thus may contribute in varying degrees to the increased thrombotic risk. Currently, there is limited molecular data available on the role of the adhesion of leukocytes in the thrombus formation of MPN. However, it appears that mutation-inherent activation of leukocyte and erythrocyte integrins plays a significant role in the prothrombotic state. This is a new angle to examine the participating factors driving thrombo-inflammation. Better understanding the potential role of integrins and involved pathways in leukocyte and erythrocyte activation may ultimately provide novel targets for prophylaxis and therapy for thrombo-inflammation in MPNs.

## Figures and Tables

**Table 1:** Up-regulation of inflammatory cytokines in MPN (inspired by [13,20,24,25]).

		References		
Cytokine	Main Function	PV	ET	MF
IL-1β *	Pro-inflammatory, acute phase, stimulating TH/B-cells, proliferation, apoptosis, and differentiation	[26,27]	[26,27]	[21,26,27,28,29,30]
IL-1Ra *	Blocking IL-1	[27,28]	[27]	[21,27,28,31]
**IL-5**	Growth factor of eosinophils, enhancing B-cell proliferation, and antibody production	[26,27,28,29]	[26,27,28]	[26,27,28,32]
IL-6 *	Pro- and anti-inflammatory, acute phase, differentiation, and cytokine production	[26,27,28,29,33,34]	[26,27,29,33,34]	[21,26,31,33,34]
IL-8 *	Chemotaxis, activating and degranulating neutrophils, and angiogenesis	[27,28,29,33,35]	[27,29,33]	[21,27,31]
**IL-10**	Anti-inflammatory and inhibition of pro-inflammatory cytokines	[26,27,32,33]	[26,29,33]	[21,26,27,28,29]
TNF-α *	Pro-inflammatory, acute phase, cytokine production, proliferation, and apoptosis	[26,27,29,33,36]	[26,27,29,33,36,37]	[21,26,27,28,30,31,36,37]
IFN-α *	Anti-viral	[26,27]	[26,27]	[21,26,27,28,29]
IFN-γ *	Promoting T_H_1 and the cellular immune response and activating macrophages	[26,27,28,33]	[27,29,33]	[26,27,30,31]
TGF-β *	Inhibiting growth, activating leucocytes, inducing T_Reg_, apoptotic, antiangiogenic, and healing wounds	[29,38]	[29,38]	[38]
**VEGF**	Vascular growth factor: vasculogenesis and angiogenesis	[27,28,29,33,39]	[27,29,33,39]	[21,27,28,31,32,39]
CCL2 (*) (MCP-1)	Recruiting monocytes, activating macrophages, histamine release of basophilic cells, stimulating T_H_2	[28,33]	[27,29,33]	[21,27,29]
**CCL3 (MIP1-a)**	Stimulating T_H_1 and DC	[26,28]	[26]	[21,26,28,29,31]
**CCL4 (MIP1-b)**	Stimulating DC	[26,27,28]	[26,27]	[21,27,29,40]
**CXCL9 (MIG)**	Activation of the acquired immune system	[27,28,32]	[27,32]	[21,27,28,29,32]
**CXCL10**	Pro-inflammatory, anti-angiogenetic, and stimulating T_H_1	[26,27,28,32,36]		[21,26,27,28,29,36]

Summary of the most described up-regulated cytokine levels in the peripheral blood of JAK2-V617F-positive polycythemia vera (PV), essential thrombocythemia (ET), and myelofibrosis (MF) patients compared to healthy controls. In a recent review, it was mentioned that “a useful correlation of cytokine profiles with driver mutations is currently impossible to generate, since only scattered and not univocal associations have been reported” [20]. This suggests that “somatic mutations in the MPN clone are not the only determinant of the MPN inflammatory state” [20]. * Evidence for involvement in thrombus formation [28,41,42,43].

## Data Availability

Not applicable.
